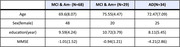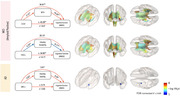# Mediation of Physical Activity Patterns between Whiter Matter Microstructure Alteration and Cognitive Decline According to the Progression of Alzheimer's Disease

**DOI:** 10.1002/alz.091337

**Published:** 2025-01-09

**Authors:** Narae Kim, Hyun Woong Roh, Sang Joon Son, Bumhee Park

**Affiliations:** ^1^ Ajou University School of Medicine, Suwon, Gyeonggido Korea, Republic of (South); ^2^ Ajou University Medical Center, Suwon, Gyeonggido Korea, Republic of (South)

## Abstract

**Background:**

We aimed to clarify the role of physical activity patterns on cognitive function decline caused by white matter structure alteration following the progression of Alzheimer’s disease(AD). Physical activity has been reported as having a close association with various pathological factor of dementia, especially it has been proved as a protective factor of disease advancement. Comparing the mediating models between Mild Cognitive Impairment (MCI) and AD, we aspire to elucidate how activity patterns, as a biomarker, undergo progression in the pathogenesis of the disease.

**Method:**

Data from Biobank Innovation for chronic Cerebrovascular disease With ALZheimer’s disease Study (BICWALZS, N=131, 93 females, age = 71.66 ± 7.51) were used and we divided the cohort into three subtype groups of dementia; MCI with Amyloid beta negative(MCI‐), MCI with Amyloid beta positive(MCI+) and AD. Fractional Anisotropy (FA) was extracted from 48 regions as defined at JHU atlas and 6 activity pattern parameters (L5, M10, IS, IV, kRA, kAR) were calculated. Mediation analysis was conducted regional FA values as a predictor, a standardized score of Mini‐Mental State Examination as a response variable and the physical activity parameters as a mediator for each group separately.

**Result:**

We found significant mediation effect in the MCI+ group after adjusting age, sex, and education year (FDR corrected P < 0.05). 20 white matter regions showed significant mediation effect with mean physical activity intensity during most active 10 hours (M10) and 24 regions showed significant mediation effect of Intraday variability (IV). Although there was similar result in AD group, but the only one region showed, and the effect size of mediation path was quite small. There was not any significant result in the MCI‐ group.

**Conclusion:**

Summing up, these results can be interpreted as that the protective effects of physical activity on the cognitive decline caused by white matter alteration is far larger to patient with amyloid deposition and the effects can be decreased after the onset of AD. Therefore, enhancing physical activity patterns can be an effective strategy to slow down the progression of the disease, particularly in amyloid‐positive patients, prior to the onset of AD.